# Informing the development of online weight management interventions: a qualitative investigation of primary care patient perceptions

**DOI:** 10.1186/s40608-018-0184-6

**Published:** 2018-02-12

**Authors:** Samantha B. van Beurden, Sally I. Simmons, Jason C. H. Tang, Avril J. Mewse, Charles Abraham, Colin J. Greaves

**Affiliations:** 1 University of Exeter Medical School, University of Exeter, Exeter, UK; 2 Psychology, University of Exeter, Exeter, UK; 3 School of Medicine, University of Dundee, Dundee, UK

**Keywords:** Weight loss, Obesity, E-health, Internet, Primary care, Qualitative research

## Abstract

**Background:**

The internet is a potentially promising medium for delivering weight loss interventions. The current study sought to explore factors that might influence primary care patients’ initial uptake and continued use (up to four-weeks) of such programmes to help inform the development of novel, or refinement of existing, weight management interventions.

**Methods:**

Semi-structured interviews were conducted with 20 patients purposively sampled based on age, gender and BMI from a single rural general practice. The interviews were conducted 4 weeks after recruitment at the general practice and focused on experiences with using one of three freely available weight loss websites. Thematic Analysis was used to analyse the data.

**Results:**

Findings suggested that patients were initially motivated to engage with internet-based weight loss programmes by their accessibility and novelty. However, continued use was influenced by substantial facilitators and barriers, such as time and effort involved, reaction to prompts/reminders, and usefulness of information. Facilitation by face-to-face consultations with the GP was reported to be helpful in supporting change.

**Conclusions:**

Although primary care patients may not be ready yet to solely depend on online interventions for weight loss, their willingness to use them shows potential for use alongside face–to-face weight management advice or intervention. Recommendations to minimise barriers to engagement are provided.

**Electronic supplementary material:**

The online version of this article (10.1186/s40608-018-0184-6) contains supplementary material, which is available to authorized users.

## Background

Overweight and obesity remain a worldwide problem. Guidelines from the National Institute for Health and Care Excellence (NICE) recommend that GPs monitor patient weight and offer clinical weight management where necessary [1], but the choice of treatments in traditional primary care settings is limited. Currently, patients are offered lifestyle modification, pharmacotherapy (Orlistat), or weight loss surgeries of which the latter two are effective but expensive and often accompanied by negative side effects or complications [2, 3]. Referral to community-based weight loss programmes has been shown to be effective in the short term [4, 5], but resources are limited and face-to-face programmes can be costly to implement [6] and limited access restricts their use in rural areas [7]. Moreover, research suggests that primary care staff feel under-resourced to provide weight loss services [8, 9] and practitioners seldom approach the topic of weight [10–13].

Self-directed interventions delivered via digital platforms (eHealth) are plentiful and could provide a low cost and easily accessible alternative to existing treatment options in primary care [14, 15]. Such interventions can range from educational websites focused on information provision such as NHS Choices, to the more intensive mobile applications that offer interactive food diary and weight monitoring tools such as MyFitnessPal. With 87.9% of adults in the UK using the internet and the 68.7% increase in prevalence of recent internet use among adults aged 65 to 74 (currently at 74.1%) between 2011 and 2016 [16], these interventions have the potential of reaching a substantial proportion of the UK population. E-Health has been found to be effective for a range of health behaviours including smoking, reducing cholesterol levels, lowering high blood pressure [17, 18], and facilitating weight loss [14, 19]. Technological advances have enabled the delivery of behaviour change techniques that map on to theoretically derived behavioural determinants, which were previously limited to face-to-face delivery such as prompting goal setting, instructing progress monitoring, and providing timely goal-related feedback [20] and are associated with effectiveness in face-to-face weight loss programmes [21]. In addition, service providers are positive about the use of eHealth in terms of its potential to provide continuity of care and opportunities for auditing the provided service [9].

However, not much is known about the experiences of primary care patients with eHealth for weight loss or their willingness to use it when recommended by their GP. This applies particularly to middle-aged and older patients living in rural areas with limited or no access to traditional weight management. Therefore, this study aimed to investigate the experiences of rural primary care patients with GP-facilitated use of such programmes and the factors that may influence their adoption and ongoing use to help inform the development of a novel weight management interventions.

## Methods

### Study design and setting

A qualitative study was conducted, using face-to-face semi-structured interviews with patients from a single General Practice in rural South West England. Ethical approval was obtained from the East of England Research Ethics Committee (Norfolk) in November 2012. No predetermined theoretical framework was used to develop the design of the study.

### Weight loss websites

Three freely available weight loss websites were selected for use in this study to access a range of experiences to help identify facilitators and barriers to the use of publicly available internet-based weight management programmes. The selection of these websites was based on the additional criteria concerning their compatibility with current US and UK recommendations for healthy eating as checked by a GP (SS):

1. SparkPeople (http://www.sparkpeople.com/) is an online community which requires registration and profile creation. The website provides diet tips & coaching from nutritionists and other experts; healthy recipes; exercise instructions in educational videos; food and weight monitoring tools; visual feedback on progress and goals; access to support via the internal social network such as the forums and blog options as well as the opportunity to link out to personal social media such as Facebook.
2. LiveWell (https://www.nhs.uk/livewell/loseweight/Pages/Loseweighthome.aspx) is part of the NHS Choices website and provides access to a 12-week diet and exercise plan in PDF format that can be printed off and completed by the individual; other information available on the website are healthy recipes; information about BMI and calorie counting; large collection of links out to various related useful information sources and dieting tools.
3. LiveStrong (http://www.livestrong.com/) is an online community which requires registration and profile creation; this website gives access to educational exercise videos; healthy recipes; and information and tools for calorie goals and counting, as well as weight progress monitoring and access to social support via the community forum.

### Participants

Participants were identified by a GP (SS) through (a) a single General Practice patient database search and (b) opportunistic recruitment during consultations at that same General Practice situated in rural South West England from which patients are on average older than in the rest of the UK (26.9% aged 65+ vs 17.2%), predominantly Caucasian (99.5%), and living in an area that is among the 40% least deprived (IMD 12.8) [16, 20]. Searches were conducted using the inclusion criteria in Table 1. Of the 649 eligible participants, 64 were invited using a purposive sampling framework [22] to select for diversity in terms of gender and BMI.

**Table 1:** Inclusion and exclusion criteria

*Inclusion criteria*:
(1) desire to lose weight
(2) aged 35–60
(3) BMI of 30 to 45 kg/m^2^
(4) able to access to the internet via a computer or other device.
*Exclusion criteria*:
(1) medical-conditions, such as coronary artery disease, type I diabetes or insulin-treated type 2 diabetes, stroke or cognitive impairment, terminal illness
(2) unable to read, write, or understand English
(3) learning difficulties
(4) taking medication that could affect weight.

**Fig. 1:**
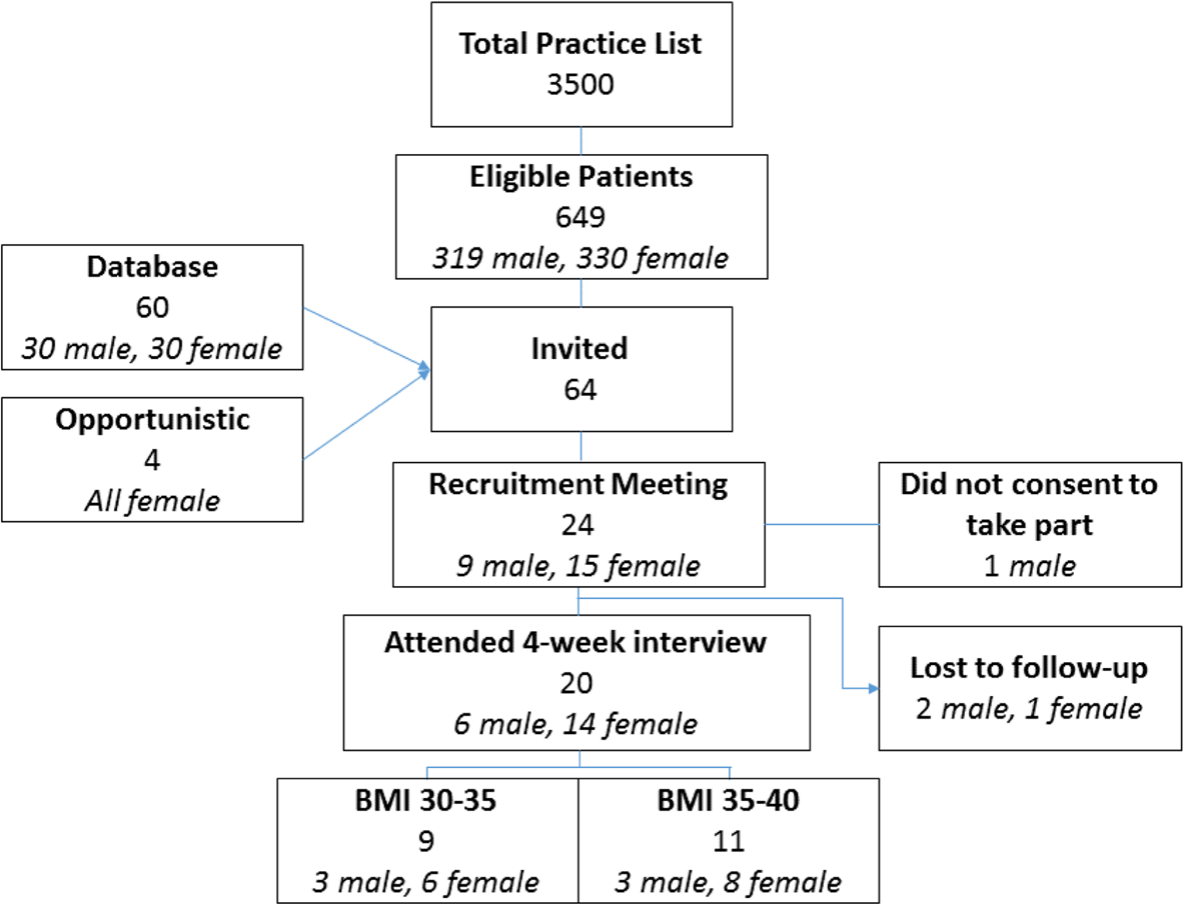
Participant identification, recruitment, and follow-up

A total of 24 participants agreed to take part. Participant identification, recruitment and follow-up can be found in Fig. 1.

### Materials and procedures

Eligible participants attended a 20-min recruitment meeting at the surgery with the GP (SS). The study was discussed, written informed consent was requested and participants were asked to complete a questionnaire providing baseline information on BMI and age. Participants were then provided with the three web-addresses (as above), asked to explore each and select their preferred choice for the following 4 weeks. The meeting ended with a 30-min semi-structured lifestyle consultation (see Additional file 1 for consultation guide).

After 4 weeks semi-structured face-to-face interviews were conducted by two female researchers (SS the GP and SvB an MSc student at the time; both attended a University of Exeter Qualitative Interviewing and NVivo course) at the General Practice Surgery, using a topic guide (see Additional file 1) which was adapted after the first few interviews. Interviews lasted approximately 30 to 50 min. Participant checking was conducted during the interview to ensure understanding of the data. The topic guide was adapted from the one used by Tang and colleagues [23] that helped explore the views of young adults (19-33 yrs) on similar websites. The interviews were audio-recorded and transcribed verbatim.

### Data analysis

Two researchers (SS & SvB) conducted inductive thematic analysis as described by Braun and Clarke [24] of the transcripts, to identify overarching themes and create hierarchies of thematic categories and presenting a realist account of the data. Using constant comparison techniques, themes were updated iteratively as new data came in. Coding continued until data saturation was reached (i.e., last 5 transcripts did not add substantially to the thematic framework). We constructed explanations of the data both within-cases (representing the patient’s journey through the intervention process) and between-cases (to identify themes that were common or divergent between people preferring different websites). We used NVivo 8 to organise the data. Thematic analysis is widely used in similar exploratory studies [25, 26].

After initial independent reading and coding of a sample of the data (10 transcripts) the two researchers noted and discussed preliminary themes in a draft coding framework. Coding of the remainder of the data continued independently. Any new emerging themes on the remainder of the transcripts were captured in an “other” node which were discussed by four members of the research team along with the existing themes in subsequent meetings (SS, SvB, AM, and CG).

## Results

Of the 24 participants recruited in the initial meeting, 20 (70% female) took part in the follow-up interview (See Fig. 1. above for participant flow). Participant characteristics and website choices are shown in Table 2.

**Table 2:** Participant characteristics and website choice

	Men	Women	All
Age	35–57	40–57	35–57
	(M = 47.3)	(M = 51.4)	(M = 49.5)
BMI	32–44.8	30.4–43.4	30–47.8
	(M = 36.2)	(M = 35.7)	(M = 35.9)
Website Choice			
Livestrong	2	1	3
LiveWell	2	6	8
SparkPeople	2	7	9

All participants were familiar with the use of the internet and selected their preferred website to continue with. Only seven participants indicated they were either still using their chosen website or aimed to continue using it at the four-week interview. However, some of those who were no longer using their website reported they would be interested in using a different website providing it encompassed some of the suggestions they provided (as below).

The key stages of our participant journey in engaging with weight management websites form the four overarching themes (Table 3) and provide the framework for structuring our analysis and subsequent results section. Each of these overarching themes comprises several sub-themes and these are described and evidenced in detail. Organising the results in this order will allow exploration of facilitators and barriers at each stage which may help inform ways of minimising programme drop-out and nonadherence to optimise potential effectiveness. Some similar subthemes weave across various stages, these are highlighted as facilitators and barriers in Table 4 with reference to the respective stages.

**Table 3:** Key stages of patient journey

(1) initial interest and website choice
(2) engagement with and use of the website
(3) implementation of changes
(4) continued use of the website and behaviour change. Within these stages a number of themes were identified.

**Table 4:** Main facilitators and barriers identified across the key stages

Facilitator/Barrier to website use	Influencing factor
Facilitators	(1) Motivation (Stage 1–4); from Tracking features and Email reminders.
	(2) Personal preferences (Stage 1&2); Appeal of website; Email reminders
Barriers	(1) Effort (Stage 2–4); Time and commitment (Stage 3)
	(2) Lack of novel or useful information (Stage 4)
	(3) Accessibility and disposability; (Stage 4)
	(4) Email Reminders (Stage 2)
	(5) Perceived target group (Stage 4); Appeal of website (Stage 1)

### Stage 1: Initial interest and website choice

#### Motivation

Participants were motivated to lose weight to improve physical appearance, self-confidence and health consequences, and due to feelings of obligation to others. For example:

“*I have a BMI of 41, I have to find a way of conquering that. My daughter is graduating in September and I’d love to be a stone and a half lighter, y’know I have got goals.”* (Participant 1, F, 57)

“*I wouldn’t say life expectancy is an issue that drives me erm I will live however long I live … however I think the key things are how I feel and how I look. They're the motivation factors.”* (Participant 17, M, 48)

It is important to note that some participants credited the initial GP recruitment meeting for motivating them to try out the websites for weight loss.

#### Internet as a source of information

Participants described regularly using the internet to look up topics of interest and this may have facilitated their willingness to try online weight loss programmes. Few participants had prior experiences with eHealth and none had heard of the three websites offered in the study. Their choice of website was influenced by the website’s ability to satisfy their information needs about weight loss and its potential health consequences. For example:

“*That’s the one that seemed to have got more of the information that I was looking for really… I like the fact they send you, you have lots of recipes y'know so it's sort of easy then to buy ingredients and to put together meals er it just, it just seemed to me to be the one that best suited me really*.” *(Participant 20, F, 47 years)*

#### Appeal of the website

Participants wanted the amount of information provided to be satisfactory and relevant but not overwhelming or bombarding as this could result in difficulties with navigating the website itself.

“*Just a lot of information to go through I didn’t find them, any of them actually particularly easy to move through*.” (Participant 20, F, 47 years)

Only three participants chose the LiveStrong website and said that this was to try something new. The other participants felt that LiveStrong was not aimed at them and chose either SparkPeople or LiveWell.

“*I’m an overweight, middle-aged woman and it seemed to me that this was geared up to young, fit people who wanted to exchange views about being even younger and fitter*.” (Participant 9, F,49)

The features that were appealing to those choosing one of the latter two websites, seemed to be the exact features that led to dismissal of that particular website for the others.

“*…if you can imagine a glossy magazine that you buy… it was very much like that, but on the website* [SparkPeople] *instead of a magazine. So I felt involved, I felt invited and I wanted to interact more with it.”* (Participant 17, M, 48 years)

*“Well the American websites* [SparkPeople and LiveStrong] *just seemed a bit blotchy… it’s like when you watch shopping channels it was like trying to sell you something and I didn’t like it…*” (Participant 18, M, 35 years)

Although personal preferences influenced the initial choice of a website, the overall appeal of weight loss websites seemed to be increased by having a clear structure, ease of navigation, and being relevant to the target audience.

### Stage 2: Engagement with and use of the website

#### Tracking features

Most participants appreciated and enjoyed using the self-monitoring features such as food and activity trackers.

*“…they track you… I also find that doing that made you want to eat more healthily, because you want to come in under… it’s probably a human nature competitive y’know… I can beat this.”* (Participant 12, M, 50 years)

Participants using these tools seemed to gain motivation from the continuous reflection involved in monitoring their goals and lifestyle changes. The tools highlighted the discrepancy between current behaviour and their goals, showing what changes worked for them and which did not.

“*it is like a sort of working document that you can update regularly and just having something like that is in itself a motivation because you’re reminding yourself of something you wanted to do, you know, maybe I set a target a month ago and as I’m getting older my memory’s not so good anyway, I can’t remember what I’ve done, but I can look back, oh yes that’s what I said I’d do then, review it now, review it again in a month’s time, change it, adapt it and keep it moving on so it’s the sort of continuous reflection and making yourself decide what your priorities are and where you want to be in three, six months’ time*” (Participant 05, F, 54 years).

Although most participants thought it was easy to use the tools, by the end of the 4 weeks all participants reported that it had become too much of an effort. This was especially true for food trackers which required accurate logging of ingredients and quantities.

“*But you put in something like potatoes or something, mash potato and then they’d come up with a whole great long list of mash potato,… then it gave you how many ounces or whatever like that, so you had to be really thinking all the time*.” (Participant 15, F, 57 years)

Some mentioned that they would have liked some sort of useful feedback on or reward for their progress and this was suggested as a possible way of minimizing the perceived effort*.* With regards to the food tracking features, some participants showed an aversion to the “*old message of calorie counting*” as “*I know it doesn’t work for me*” (Participant 3, F, 56). In some cases a boomerang effect of calorie counting was also referred to. When the food tracker showed the intake to be below the daily target*,* this prompted some to eat more to make up the missing calories. This was also mentioned in relation to using calories burned through exercise as an excuse to allow increased eating.

“…*oh I’m doing all this exercise; I can eat all this food*.” (Participant 23, F, 40 years)

#### Email reminders

Both SparkPeople and LiveStrong send out regular emails including weekly newsletters, health tips, and tracking (self-monitoring) prompts. In some cases the abundance of emails led participants to avoid the website. For example:

*“I see Sparkpeople Sparkpeople… Sparkpeople, and I think OH, and walk away.”* (Participant 11, F, 56 years)

However, other participants enjoyed reading these emails and accredited their ongoing use of the website to them.

“*They actually sort of instigated my use of their website by these emails, ok? and I find that kept me going back, I mean, other than that, I probably wouldn’t have gone there so often.”* (Participant 12, M, 50 years)

This variability in responses to emails shows the strong influence of personal preferences on engagement with online weight loss interventions. This is a common subtheme that cuts through various stages, and was previously encountered in Stage 1 where personal preferences influenced initial use and website choice (See also Table 2). Importantly, even those who did not like the emails mentioned they did not wish to entirely cut them.

“*I wouldn’t want more than an email every couple of weeks really, I find it really off putting that they just email and email and email, but the occasional email, yes, if it’s got some pertinent information on it*.” (Participant 09, F, 49 years)

#### Social networking in relation to weight loss

All three websites provide access to a social support system in the form of internal online forums, linking to existing forums, or linking to the user’s Facebook account allowing progress to be posted onto their personal profile. Although participants thought these forums were helpful for gathering information, they showed an aversion to engaging interactively. None of our participants actively posted in the available forums or uploaded their progress to Facebook.

“*You can read all these other people’s comments which are quite helpful but I certainly wasn’t gonna put anything about myself on there...”* (Participant 10, F, 54 years)

### Stage 3: Implementation of changes

#### Time and commitment

The time taken to fully engage with the websites’ features and implement the encouraged lifestyle changes was a substantial barrier.

“*I s’pose you have to do it like that to be accurate but I haven’t got enough hours in the day I’m afraid to weigh my mash out*.” *(Participant 15, F, 57 years)*

Some participants mentioned that to commit to the lifestyle changes and the time needed to fully use the website, these changes would ideally need to feel effortless or they need to be more convinced that making these changes would guarantee weight loss. .

“*I’d like just something with simple recipes on it, and a choice of recipes for like, for a whole week, with simple ingredients that you know, you could, you would know, that if you followed this, this plan for a week, erm, bit like Weight Watchers but, if you, if you followed this plan for a week, you would definitely lose weight and if you did exercise as well*.” (Participant 11, F, 56).

#### Translating motivation into action

Most participants reported being able to successfully integrate small changes into their lives, such as walking to work instead of driving and joining -grandchildren, partner, clients, or dog- for walks’ and changing snacking habits. Some of these changes were attributed to the information provided on the websites or community forums, for example:

*“…the good thing from it is I've changed a lot of habits… I really have changed… It's just about thinking before you put it in your mouth sort of thing you know. I've not, this is a shocker, I've not touched a soft drink since looking, I just drink sparkling water now. Probably 4 or 5 pints a day which is a shock for me. So yeah it's sort of changed me a little bit as well cos when I first looked at it me and my wife, cos it's telling you about sugars.”* (Participant 18, M, 35)

In some cases successful implementation of changes was facilitated by other behaviour change techniques such as the need to record behaviour.

“*I’ve been more conscious of what I’ve been eating and again a sense of guilt because if you have to record it.”* (Participant 5, F, 54 years)

Making small and manageable changes also seemed to be associated with a higher chance of success. For example:

“*I’ve been eating a bit less but I haven’t changed what I eat, I’ve just been eating a little bit less and it hasn’t taken me an awful lot of effort and I think I have lost some weight according to my own scales and that’s fine, I’m quite happy with that.”* (Participant 16, M, 55)

The same was found for changes in physical activity. In contrast, the promotion of intense physical activity was reported to be off-putting.

“*The two things that I've tapped into are the walking one because that’s what I can, I like to walk, I've always liked to walk, I also walk for a purpose cos I gotta take my dog places. I can even measure the distance I have to walk from the house to the bus stop so that was very practical.”* (Participant 19, M, 50)

Although participants credited their initial motivation to GP (Stage 1), they also wanted further facilitated support alongside internet-based weight loss programmes used in primary care settings in the form of face-to-face meetings with a health professional, as this would give them an extra push to make lifestyle changes.

*“…to be totally honest, the whole thing, just the talking with you [the GP: SS] was just amazing, but that really, and as I now hate groups, it’s actually nice talking to a real person, than it is to looking at a website. I think you’d have to have a combination of the two*.” (Participant 10, F, 54 years)

### Stage 4: Continued use

#### Lack of novel or useful information

The participants reported going back to the website as long as the information was still considered to be new and helpful. However, most participants mentioned that they felt the information was often just the same as everywhere else.

“*Maybe I’m too simplistic, but it all basically comes down to eating less and exercising more and there’s only so many people that you can listen to telling you that…” (Participant 24, F, 52 years)*

They mentioned that instead of being told what to eat or not eat (which they already knew), they needed help with, or strategies for dealing with temptations or the pressure to revert to old habits to eat unhealthily in various situations such as when under time pressure, in social situations, or when confronted with tempting foods.

“*you can be motivated but in all the different points during the day where there is the option to, every time again you have to make the choice, shall I or shan’t I?... So you're constantly confronted with it as well.”* (Participant 24, F, 52)

*“I’m trying to feed the family with things that they normally like and they’re very entrenched in set behaviours and my husband does the shopping and it’s quite difficult to get him to buy different things*.” (Participant 5, F, 54)

#### Effort

Another reason people gave for not maintaining changes was that the intervention methods promoting them were considered to be arduous and too much of a hassle for continued use. This was particularly true when monitoring efforts involved weighing of food*,* converting measurements, logging of eating and exercise behaviours*,* and reading emails. Some participants remarked that integrating the websites with a smartphone app might be a way to minimise the effort involved in engaging with certain behaviour change techniques.

“… *if you wanted to make a food diary then you’ve got to keep logging back in and it would be much easier to have something maybe by your side, that you could use almost, on your phone or something like an app.” (Participant 20, F, 47 years)*

#### Accessibility and disposability

Continued use of the websites was also influenced by the way participants accessed the internet in day-to-day life. Although all participants were confident computer users, some reported a lack of interest in using them in their leisure time. They considered these programmes to be more beneficial for those who ‘*love being on the computer’ (Participant 11, F, 56).* Some also hinted at the disposability of weight loss websites - they are easily closed and soon lose their novelty value.

“*No staying power… I just get bored of this all eventually, move on to the next exciting thing…”* (Participant 17, M, 48 years)

Smartphone apps were mentioned again as a way to improve accessibility.

“*If you had a smartphone and you used one and it was beside you when you were cooking or something, it might be more intuitive to do it that way because it’s with you all the time.”* (Participant 15, F, 57)

#### Continued motivation

Although the participants were recruited based on their desire to lose weight, their comments suggested a loss of motivation over time. A greater emphasis on health risk information was suggested as a means of increasing motivation. For example:

“*I don’t want them saying to me, you should eat less fat and you should eat more of this and less of that because I already know that. What I think they should be doing is saying, if you’re more than 10% overweight or whatever it is then you’re putting yourself at quite a big risk of diabetes, this is what happens to you if you get diabetes, and that was sort of missing I felt*.” (Participant 16, M, 55)

However, for some, information on long-term health consequences did not feel like an imminent threat. Those who perceived the problem to be more urgent reported being more vigilant in terms of adhering to the lifestyle changes.

“*Somehow even threats of diabetes and goodness knows what…it’s perhaps not immediate enough.”* (Participant 24, F, 52 years)

“*some days I have said no to eating something ‘cause I do know that this is the long haul so I’m quite excited that I’m just going to keep going… I just know I’ve got to, I can’t give up now ‘cause of my health so I’ve just got to keep going*.” (Participant 10, F, 54)

#### Perceived target group

Finally, the matching of the website content to individual preferences is not only important to initial uptake, but also crucial to continued use. Although all three websites were aimed at people who wanted to lose weight through diet and exercise, some participants still felt the websites were ‘*too young’* and ‘*too modern’ (Participant 11, F, 56)* or that the website wasn’t quite aimed at them which particularly hindered continued use. For example:

“*…It [LiveStrong] was certainly more for a sort of like bodybuilder, and I wasn’t going to do bodybuilding… that was the impression that I got, and that was why I didn’t really use it, because it just didn’t feel like it was pertinent to me and what I wanted.”* (Participant 12, M, 50 years)

## Discussion

### Summary

This study explored primary care patients’ experiences of using weight loss websites facilitated by a single brief contact with a GP. A number of facilitators and barriers to website use were identified (See also Table 4). Website components that were considered facilitative by some participants were seen as barriers by others (e.g., multiple email reminders). In addition, features that facilitate initial engagement may discourage continued use over time (e.g., food logs). These individual differences in the appraisal of components highlight the need for personal tailoring in online weight loss websites. The data driven recommendations for eHealth selection and development for use in primary care settings are summarized in Table 5 which have helped inform the development of a novel smartphone app-based weight management intervention focused on the modification and management of nonconscious processes to facilitate dietary change.

**Table 5:** Recommendations for future development and refinement of internet-based weight loss interventions

1. Future internet-based interventions that are designed to facilitate weight loss consultations given in primary care settings should be personally tailored where possible, to allow for choice of style (e.g. technical, health-focused) and delivery formats (e.g. internet, smartphone), and ideally allowing users to adjust the number of reminders to prevent users from feeling harassed.
2. To maintain interest, content and features need to be novel (e.g., temptation resistance strategies) and updated, yet require very little effort from the user to find and use (i.e. good organisation of detailed information allowing users to find what they want easily).
3. Tracking features should be appealing and require less effort from the user than current methods (e.g., use of smartphone barcode scanners, auto-tracking of activity using devices, tracking weight or success with planned changes, rather than total calories consumed).
4. Lifestyle changes should be presented in a manner that reduces the perceived effort and time to implement such changes (i.e., reduce portion size vs weighing and logging every ingredient).
5. The intervention may need to address issues of motivation and prioritisation to support more resource-intensive changes.
6. Particular care is needed to ensure that social support elements of interventions provide a safe environment in which to disclose sensitive information.
7. The use of face-to-face support alongside web-based support may be advantageous when implementing internet-based interventions in primary care settings.

### Strengths and limitations

To our knowledge, this is the first qualitative investigation of middle-aged adults’ use of eHealth for weight loss in the context of primary care service delivery. The study’s strengths include use of a real-world health care context where participants were offered weight management treatment by a GP as part of their clinical care and the rigorous application of qualitative research methods. However, some limitations need to be acknowledged. Some participants acknowledged that involvement in a research study had motivated their use of the website, so the findings may not translate to a non-research context. In addition, the study used self-reported accounts of engagement with the websites and of lifestyle changes. Further research could incorporate actual website usage data and objective measures of behaviour change such as accelerometry and weight. The gender distribution (70% female) is a further limitation, restricting the range of views and experiences captured in this study. Unfortunately, the gender distribution in this study is seen in other internet-based intervention studies [14, 27] which highlights the need for further investigation of strategies to improve male engagement with, and adherence to, weight management interventions. Due to this distribution, as well as the sample size we did not explore gender (or age) differences in depth. However, no potential differences were noted during analysis. Our sample was drawn from a rural population in South West England. The findings may not transfer well to the wider rural primary care population in the UK.

### Comparison with existing literature

Previous research has shown that monitoring features are positively perceived in behaviour change interventions [28] and techniques such as self-monitoring, and goal setting are associated with effectiveness in facilitating weight loss [21, 29], although the evidence linking these change techniques to effectiveness in eHealth interventions is limited [30]. Our research shows that, although such features are often useful they may result in disengagement from the intervention if they are time-consuming.

Our participants disliked interactive use of social support tools which required disclosure of intentions and /or progress, yet evidence suggests that social support is positively associated with weight loss both in eHealth [31] and other weight management programmes [32]. Therefore, it is important to investigate strategies to enhance engagement with social support tools. However, public online platforms can be perceived as untrustworthy [23]. This suggests that additional forms of support may be required such as encouraging the (offline or online) involvement of an existing relation or friend. Age may be a factor affecting the use of these social support tools. Tang and colleagues [23] identified that young adults (age range 19–33) who are familiar with internet applications value attractive user interface, structure, ease of use, personalisation and accessibility when using eHealth for weight management. Social support tools were motivating for some, but not all. This contrasts with our findings where none actively engaged, or were interested in actively using, social networks for weight loss support. Intervention developers also need to consider the age of their target group when developing their materials. LiveStrong was particularly off-putting to our sample due to the images of young and healthy active people and therefore confusing the target population. Additional file 1: Table S1 compares the findings of Tang and colleagues [23] with the findings of the current study. This table highlights differences in views about social networking, as well as similarities in factors affecting the appeal of, and initial engagement with, weight loss websites.

Finally, personal-tailoring has been suggested to be of importance for adherence to eHealth [23, 32, 33]. The current study supports this idea, suggesting that personal-tailoring should involve allowing participants to set preferences for feedback and email frequency as well as units of measure. Tailoring content to individual motivations and barriers to change (which can be assessed by questionnaire) might also help to engage people more strongly.

## Conclusion

Although primary care patients are willing to use and engage with eHealth, they expressed a strong preference for additional support or facilitation. There is therefore potential for the use of such interventions in primary care settings alongside standard weight loss advice. This study has helped inform the development of a smartphone app-based intervention that provides strategies and in-the-moment support to help individuals resist food-related temptations in order to facilitate weight loss. It is crucial to identify or develop weight management eHealth options that enhance the facilitative features and minimize barriers identified in this study. Although this does not guarantee treatment effectiveness, ignoring such changes may result in non-use and therefore ineffectiveness.

## Additional file

Additional file 1: Consultation guide, interview topic guide, and comparison of findings with existing literature. (DOCX 27 kb)

## Abbreviations

eHealth: Electronic Health
GP: General Practitioner
NICE: National Institute for Health and Care Excellence
