# Supplementary material for: Informing the development of online weight management interventions: a qualitative investigation of primary care patient perceptions
Source: BMC Obes. 2018 Feb 12;5:7. doi: 10.1186/s40608-018-0184-6 (PMC5810085; doi:10.1186/s40608-018-0184-6)
Supplement: Supplementary file 1 — Consultation guide, interview topic guide, and comparison of findings with existing literature. (DOCX 27 kb) [file 40608_2018_184_MOESM1_ESM.docx]

# Supplementary File for the article “Informing the development of online weight management interventions: A qualitative investigation of primary care patient perceptions.”

Samantha B van Beurden, Sally I Simmons, Jason C H Tang, Avril J Mewse, Charles Abraham, and Colin J Greaves

The main paper describes the qualitative investigation into factors affecting primary care patient engagement with commercially available online weight loss programmes following a brief lifestyle consultation with the general practitioner, highlights the themes identified, and offers recommendations based on the study’s findings. This supplement contains detailed information illustrating the lifestyle consultation provided, the interview topic guide used, and Table S1 highlights the experiences of online weight loss interventions in different contexts.

*Press Ctrl+Home to get back to this page.*

| **Table of contents** | |
| --- | --- |
| 30-minute semi-structured lifestyle consultation | [2](#lifestyleconsultation) |
| Four-week follow-up interview topic guide | [4](#topicguide) |
| Table S1: Experiences of online weight loss interventions in different contexts | [5](#tableS1) |
|  |  |

**30-minute semi-structured lifestyle consultation**

**Would you mind if we spent a few minutes talking about weight?- . If they say they would mind then say “that’s fine – I’m not here to nag you about it – we don’t have to discuss it – it’s entirely up to you”. Give standard advice. Not entered into study.**

**What do you know about the relationship between your weight and your health? Ask-tell-Discuss. (find out what they know, introduce some info of your own (would you be interested in this from a doctor’s point of view?) Discuss with them –“what do you make of that?”**

(Prompts – knowledge of BMI, chronic cardiovascular disease, stroke, Diabetes, MI etc. Fill in knowledge gaps if any. Make link between lifestyle and health and own interest in them).

**What do you think caused your weight gain?**

(Prompts re pregnancy wt gain, other health issues, including depression, money, relationships, weight 10 years ago?) reflect on their responses (rpt back or rephrase what they say/check that you have understood – for instance “so it has been just going up gradually over the last 10 years?” “you are right – it’s down to what you eat and the amount of activity you do”. Could add more info here is appropriate – did you know what to put on a kg a year you just need to eat 20 calories a day more than you need – that’s like half a biscuit a day..” They may have a reasonably healthy diet but might be frustrated/confused because they don’t understand how they came to be overweight – acknowledge this

?

**What would be the benefits to you personally if you could lose some weight?**

(Prompts – how would you feel if, in two years time you were 2 stone lighter (or heavier if they don’t seem to care)? Look for positive, emotional and personal explanations - are they doing it for themselves or someone else? Reflect or reframe their responses to make positive).

**If you decided to lose 2 or 3 kilos (5 pounds) over the next few months, how confident are you that you could succeed?** – on a scale of 1-10 where 1 is not at all confident and 10 is completely confident

Explore this – why do you say 5 and not say 8? (this will elicit barriers, which you can reflect on and seek to break down or help them to think of ways to overcome), or if confidence is high “why an 8 rather than say a 5?” (this will elicit reasons for confidence)

**(if it seems to fit in) ...Tell me about your experiences of trying to lose weight in the past?** Eg Have you tried any diets or weight loss programmes, and what aspects of these programmes helped you /what stopped you succeeding?

**Make a summary** of their perceived benefits and confidence levels (along with barriers or indeed reasons for confidence if confidence is high)

**So where does that leave you?**

Prompts a) silence (let them think about it) b) what do you think you will do?

They can either say I would like to lose some weight (in which case ask them to make 2-3 small changes they could make to start off with (encourage gradual weight loss and to keep adding small changes until they get where they want tobe) OR they could say “I don’t want to change anything” – in which case say “ that’s fine – we can leave it there” – offer open door if they change their mind. OR they could say they are not sure – in which case you can either explore importance or confidence further (whichever they are stuck on) or leave it with them to think a bit on (give out some information booklets – eg BHF does some good free ones.)

Move on to talk about the study as additional help/something they may be interested in.

**Introduce the research study**

“By being here today you have shown interest in our research project. We are looking at different ways to help people lose weight and are interested in whether using websites or smart phone apps can help you. We have 3 free websites with smart phone apps for you to try and would like you to choose the one you think you will get on best with and use it for at least 4 weeks. We will then meet you again for a recorded interview here at the surgery which will last about 45 minutes to see how you got on. We will then telephone you after a further 3 months just to see how you are getting on, and this phone call will also be recorded.

We are trying to find out more about how people use these kinds of website and if they help them to lose weight. If you don’t want to get involved in the research project after all, that is perfectly fine and it wont affect your care in any way.”

**If keen to participate** - discuss consent, PIS, start date and any other questions and arrange 1/12 follow up date.

**If opts out:** offer lifestyle advice and leave door open for future support

**Lifestyle advice:**

Smoking status: Smoker/Non-smoker/Ex-smoker

Offer smoking cessation advice

Dietary advice (including fats/carbs/salt/caffeine): EatWell Plate

Offer exercise advice – 30 mins brisk walking per day minimum – tailor this to mobility levels if needed.

See resources at http://www.devonhealthandwellbeing.org.uk including ...

**Take control of Your Weight (BHF)**

https://www.bhf.org.uk/publications/view-publication.aspx?ps=1001226

**So you want to Lose Weight...For Good (BHF)**

https://www.bhf.org.uk/publications/view-publication.aspx?ps=1000807

**Guide to Food Labelling (BHF)**

<https://www.bhf.org.uk/publications/view-publication.aspx?ps=1000110>

**Four-week follow-up interview topic guide**

1.How did you get on with the websites?

- Which one did you settle on using?
- Why did you not choose the other 2 programmes? Positive or negative choice? (eg. Best out of a bad bunch? )

2.Talking now about the website you did choose

- How easy has it been to use?
- How long did it take you to learn how to use this application?
- What made you choose this one over the other 2?
- What time of day/frequency did you mainly use it?
- What aspects were not easy to use?
- If you stopped using it, what were the reasons for stopping?
- What problems did you have using the website?
- How could it work better for you? Are there any features you would like to see added?
- How long in total did you use it for?

3. Have there been any changes in your weight since using the website?

- Do you think the website helped? if so, in what way?
- Has there been any family or outside influences/support?
- Do you think the website has changed the way you think and feel about food?
- Will you be continuing with the website? Yes or No? WHY?

Table S1: Experiences of online weight loss interventions in different contexts

|  | **van Beurden et al.** | | **Tang et al. (2015)** |
| --- | --- | --- | --- |
|  | LiveWell  <http://www.nhs.co.uk/livewell>  SparkPeople  <http://www.sparkpeople.com/>  Livestrong  <http://livestrong.com/> | | My Fitness Pal  <http://www.myfitnesspal.com/>  SparkPeople  <http://www.sparkpeople.com/>  Livestrong  <http://livestrong.com/>  Calorie count  <http://caloriecount.about.com/> |
| Context | Overweight and obese adults aged 35-60 recruited from a primary care setting in a rural area. | | Young adults aged 19-33 recruited from the general population |
| **Findings** | | | |
| **Motivation** | Motivated to lose weight to achieve health benefits.  Initial motivation was not enough to sustain ongoing engagement with the intervention. | | Motivated to lose weight to enhance physical attractiveness, increase self-confidence and to feel good. Health was a motivation for those who perceived themselves as being at increased risk.  Initial motivation was not enough to sustain ongoing engagement with the intervention. |
| **Appeal of the website** | Engagement and satisfaction with the interventions and their features are affected by ease of set up and use, accessibility, attractiveness of user interface, and personal tailoring. | | |
| **Tracking features** | Websites that support tracking of behaviours were perceived to be interesting and useful for monitoring and reviewing.  However, tracking must require little effort and offer some kind of useful feedback in order to facilitate on going engagement and user satisfaction. Tracking features facilitate competitiveness but also have the potential to trigger a backlash effect (compensatory eating if calorie targets are exceeded). | Websites that support tracking of behaviours were perceived as helpful for ongoing engagement. Having a comprehensive food list providing not only estimated calorie intake but also other health-related feedback was associated with user satisfaction. Food scanners make logging of food and its nutritional components easier than entering data by hand, but may be difficult with multiple or obscure ingredients. | |
| **Email reminders** | Notifications and reminders were perceived to be useful in instigating actions in users.  However, these features can also become annoying and seen as “overwhelming” and “bombarding”. | Notifications and reminders were perceived to be useful.  This also included reminders to self-monitor. | |
| **Social networking in relation to weight loss** | There was an aversion to sharing information related to weight and health behaviour via social networks as such information was considered to be very personal. Associated forums were mainly used for observation purposes, rather than interactive use. | Some participants perceived the awareness of being monitored by peers supportive, and the online communities that allow for social comparisons enhanced their self-efficacy. However, others were less sure about sharing their progress and felt that forums and discussion were untrustworthy. | |
| **Translating motivation into action** | Regular self-monitoring increased awareness of the influence of small lifestyle choices on daily energy balance. This encouraged evaluation and adaptation of such choices and the desire to change old habits.  Advice is implemented where patient perceives changes are necessary and considers these changes to be effortless. However, changes such as ‘intense’ physical activity were off-putting. | Regular self-monitoring increased awareness of the influence of small lifestyle choices on daily energy balance. This encouraged evaluation and adaptation of such choices and the desire to change old habits. | |
| **Laborious** | The calorie counting process was considered to be laborious and unsustainable in the long term | The calorie counting process was perceived to be laborious and unsustainable in the long term. | |
